# Depth prediction of urban waterlogging based on BiTCN-GRU modeling

**DOI:** 10.1371/journal.pone.0321637

**Published:** 2025-04-23

**Authors:** Quan Wang, Mingjie Tang, Pei Shi

**Affiliations:** 1 School of Internet of Things Engineering, Wuxi University, Jiangsu, China; 2 School of Automation, Nanjing University of Information Science & Technology, Jiangsu, China; The University of Tokyo: Tokyo Daigaku, JAPAN

## Abstract

With China’s rapid urbanization and the increasing frequency of extreme weather events, heavy rainfall-induced urban waterlogging has become a persistent and pressing challenge. Accurately predicting waterlogging depth is essential for disaster prevention and loss mitigation. However, existing hydrological models often require extensive data and have complex structures, resulting in low prediction accuracy and limited generalization capabilities. To address these challenges, this paper proposes a hybrid deep learning-based approach, the BiTCN-GRU model, for predicting waterlogging depth in urban flood-prone areas. This model integrates Bidirectional Temporal Convolutional Networks (BiTCN) and Gated Recurrent Units (GRU) to enhance prediction performance. Specifically, the gated recurrent units (GRU) is employed for this prediction task. Bidirectional temporal convolutional network (BiTCN) can effectively capture the information features during rainfall and waterlogging depth by forward and backward convolution and use them as inputs to GRU. Experimental results demonstrate the great performance of the proposed model, achieving MAE, RMSE, and R^2^ values of 1.56, 3.62, and 88.31% for Minshan Road, and 3.44, 8.08, and 92.64% for Huaihe Road datasets, respectively. Compared to models such as GBDT, LSTM, and TCN-LSTM, the BiTCN-GRU model exhibits higher accuracy in predicting waterlogging depth. This hybrid model provides a robust solution for short-term waterlogging prediction, offering valuable scientific insights and theoretical support for urban waterlogging disaster prevention and mitigation.

## 1 Introduction

In recent years, with the acceleration of urbanization and the rise in extreme weather events have led to urban waterlogging from heavy rainfall emerging as one of the most serious natural disasters, causing significant economic losses to cities [1]. In recent years, with the acceleration of urbanization and the rise in extreme weather events have led to urban waterlogging from heavy rainfall emerging as one of the most serious natural disasters, causing significant economic losses to cities that can disrupt infrastructure, paralyze services, and even result in casualties. For instance, in 2012, Beijing experienced severe rainfall that affected 1.602 million people, causing 79 fatalities and direct economic losses of 11.64 billion yuan [2]. Similarly, in July 2021, Zhengzhou faced unprecedented rainfall that impacted 14.5316 million individuals and resulted in economic damages of 1142.69 billion yuan [3]. Such waterlogging events pose significant threats to urban development and the safety of residents. Early prediction of flood-prone and low-lying areas is crucial for effective risk management, emergency response, and the formulation of effective urban planning strategies. Consequently, there has been an increasing focus among researchers on urban waterlogging, employing various methodologies including hydrological methods, numerical simulation coupled with neural network methods, and data-driven methods to accurately forecast and issue warnings about waterlogging extent and depth [4].

Hydrologic and hydrodynamic models are commonly used to assess the severity of urban waterlogging. Seenu et al [5] identified peak rainfall events based on the rainfall intensity-duration-frequency relationship, employing the SWMM model to simulate the urban stormwater flooding process and evaluate the impact of different rainfall events on the flooding process. Wang et al [6] simulated urban surface flooding based on CADEIES, a two-dimensional model of meta cellular automata, for assessing urban surface flood resilience at the urban drainage catchment scale. The SWMM is the most widely used hydrological model for calculating the generation and convergence processes of surface runoff and for simulating inundated areas [7]. Hydrological and hydrodynamic models, such as these, are the primary methods for analyzing urban waterlogging risk. Although these models have good model accuracy, they typically require extensive input parameters for calibration and validation, and the long computational times demand significant resources, often falling short of the requirements for urban waterlogging emergency management [8,9]. With the development of machine learning techniques in recent years, many modeling methods coupling numerical simulation and neural networks have merged. Liu et al [10] combined LSTM neural network model with a numerical model to establish a rapid prediction method for urban waterlogging depth. Its prediction accuracy and speed are higher than that of the numerical model alone. Zhang et al [11] established an urban waterlogging risk prediction model based on the coupling of the BP neural network with the SWMM model. Their model significantly enhances computational speed—improving it by several orders of magnitude compared to the numerical model—thereby meeting urgent prediction requirements. Yan et al [12] employed a coupled neural network and numerical modeling approach that efficiently simulates and identifies high-risk areas for urban waterlogging and predicts the waterlogging depth in these areas with impressive rapidity. However, a notable limitation of these methods is that the accuracy of the neural network results heavily depends on the outcomes of preceding numerical simulations. Consequently, substantial errors in the numerical results can compromise the overall accuracy of predictions. As information and data acquisition methods have expanded, particularly with advancements in Internet of Things (IoT) technology, the deployment of sensors has become increasingly prevalent in urban environments. Many cities have established urban waterlogging monitoring stations. However, the data collected primarily reflect the real-time waterlogging depth and do not extend to future predictions. The ongoing progress in deep learning technology, especially in the fields of computer vision [13], natural language processing [14] and recommender systems [15], has spurred several studies to train models on historical waterlogging data to predict waterlogging depth. The most representative of these is the data-driven approach based on time series.

It is worth noting that time series forecasting with single and hybrid models has made some great strides in areas such as traffic flow forecasting [16], stock price forecasting [17], power load forecasting [18] and dissolved oxygen forecasting [19]. Machine learning has proven to be a powerful and advanced technique and many neural network models have been used to predict water levels [20–22]. In addition, models such as machine learning have been used to predict water flow [23], and rainfall [24], however, such traditional models usually have limited generalization capabilities. Deep learning models, on the other hand, are able to efficiently process time-series data, capture long-term dependencies, and are generally more robust and able to perform better in more complex and uncertain environments.

Consequently, an increasing number of researchers are employing deep learning technologies to investigate urban waterlogging depth prediction. Among them, a single model still has the problem of low prediction accuracy in prediction accuracy, and the emergence of a combined model is a good solution to this problem. Zhou et al [25] proposed the GBDT algorithm to model the relationship between the rainfall process and the ponding process. This model predicts the depth of ponding by inputting rainfall-sensitive indicators including rainfall amount, rainfall duration, rainfall peak, location coefficients, rainfall intensity variance, and peak multiplier, demonstrating effective predictive capabilities. Wang et al [26] used the plain Bayes (NB) and random forest (RF) algorithms to predict the flooding process of the flooding point and the flooding process of the flooding point, respectively, yielding reliable results for the NB model and accurate predictions in line with actual conditions for the RF model, thus validating the applicability of both approaches in urban flood contexts. Liu et al [27] utilized LSTM neural network for multi-step prediction of waterlogging data and rainfall data in urban flood-prone sites. Among the three models, LSTM (MSLE) has the best prediction effect. The emergence of hybrid models based on deep learning techniques brings a new way of thinking to the prediction of waterlogging depth in urban waterlogging. Yao et al [28] proposed a new method for predicting the depth of waterlogging in urban areas based on a hybrid TCN-LSTM model. The rainfall and water depth are used as training samples to effectively predict the water depth for urban waterlogging. The hybrid model based on TCN-LSTM has low complexity requires no image data, and possesses higher accuracy compared to machine learning models. Compared to a single model, its prediction performance is substantially improved. Huan [29] proposed a hybrid modeling approach based on STL-TCN-GRU to improve the accuracy of real-time urban waterlogging forecasting. Sequence decomposition, however, may result in some loss of information when processing the raw data and is more demanding in terms of data volume and computational resources.

In summary, the above single model suffers from the problems of poor generalization and insufficient accuracy, while the sequence decomposition consumes a large amount of computational overhead. The model proposed in this paper effectively solves the problems of the above models.

In this paper, a deep learning predictive modeling method based on historical data and BiTCN-GRU neural network is proposed. The method has the following features:

1. To address the challenge of limited feature extraction ability in traditional waterlogging depth prediction, this paper proposes enhancements to the GRU neural network model. The BiTCN- GRU hybrid model presented in this paper extracts feature information from rainfall and waterlogging through bidirectional time dilation convolution, both in the forward and backward directions. This approach effectively extracts detailed information regarding the changes in these processes, allowing for a comprehensive understanding of the correlations within the data. The extracted feature information is then utilized as input for the GRU, enabling further feature extraction from the input sequences for improvement.
2. The BiTCN-GRU model shows good generalization ability compared to other models, and achieves good results on several assessment metrics for both the Minshan Road and Huaihe Road datasets. In addition, the model proposed in this paper does not use the decomposition operation on data series, which saves a lot of computational overhead.
3. The BiTCN-GRU model proposed in this paper utilizes historical waterlogging data for predictive modeling. Short-term forecasting, demonstrates an effective capability to predict waterlogging depth with improved real-time performance. For long-term predictions, it also effectively forecasts waterlogging depth within a 20-minute time window.

The content of this paper is divided into four sections, which are as follows: the second section describes the data we used and its acquisition method; the third section describes the methodology used in this paper as well as the model architecture; the fourth section discusses and analyzes the results of the experiments in this paper; and the last section summarizes and looks forward to our work.

## 2 Data acquisition

The study area of this paper focuses on two roads in Ningbo City where we have deployed a Waterlogging Monitoring Station (WMS). These systems have been deployed for two years and a large amount of data has been obtained. Some of the data are shown in The WMS comprises electronic water level gauges, single-barrel rain gauges, solar panels, data acquisition units, communication systems, and specific batteries. The primary purpose of the WMS is to gather real-time, high-precision data on the depth of urban waterlogging, and they have been strategically installed in areas susceptible to waterlogging. In our experiments, the sampling period is set to 5 minutes, allowing for timely and effective data collection. The WMS periodically transmits the waterlogging depth and rainfall data to the server using wireless IoT technology. The WMS operates as an application program that obtains the waterlogging depth and rainfall data at each point through a call interface.

Table 1 The WMS comprises electronic water level gauges, single-barrel rain gauges, solar panels, data acquisition units, communication systems, and specific batteries. The primary purpose of the WMS is to gather real-time, high-precision data on the depth of urban waterlogging, and they have been strategically installed in areas susceptible to waterlogging. In our experiments, the sampling period is set to 5 minutes, allowing for timely and effective data collection. The WMS periodically transmits the waterlogging depth and rainfall data to the server using wireless IoT technology. The WMS operates as an application program that obtains the waterlogging depth and rainfall data at each point through a call interface.

**Table 1:** Waterlogging depth data come from WMS.

Time	Rainfall(cm)	Waterlogging Depth(mm)
2020-9-19 3:22	0.8	0
2020-9-19 3:27	1.6	0
2020-9-19 3:32	1.2	20
2020-9-19 3:37	0.4	30
2020-9-19 3:37	1	30
2020-9-19 3:37	0	20
2020-9-26 5:32	4.2	0
2020-9-26 5:37	1.6	60
2020-9-26 5:42	0.4	60
2020-9-26 5:47	1.6	50
2020-9-26 5:52	0.4	70
2020-9-26 5:57	0	60

## 3 Methodology

This section describes our BiTCN-GRU model in detail. First, we describe the content of the entire forecasting modeling process in Section 3.1; we introduce the relevant elements of BiTCN in Section 3.2; we introduce the relevant elements of the GRU model in Section 3.3; and finally, we elaborate on the sliding window methodology used in this study’s time series in Section 3.4.

### 3.1 The design of BiTCN-GRU prediction model

**Fig 1:**
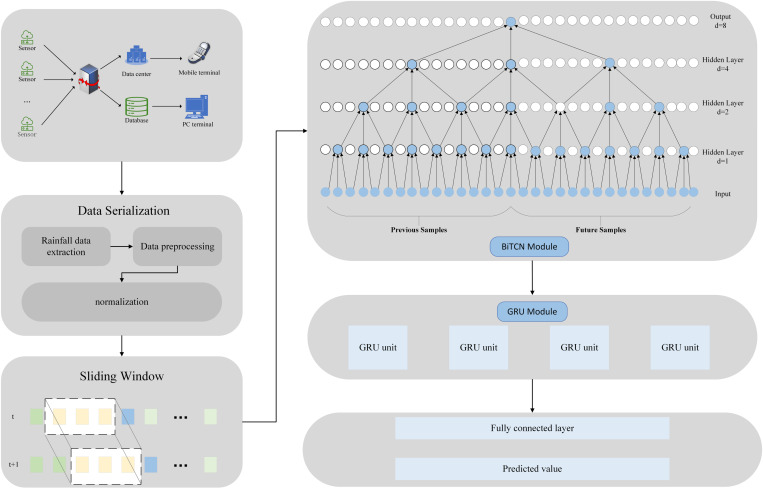
Flowchart of waterlogging depth prediction based on BiTCN-GRU model (Includes BiTCN and GRU model components).

The entire waterlogging depth prediction framework based on the BiTCN-GRU model proposed in this paper is shown in **Fig 1**.

The BiTCN-GRU model consists of an input layer, four BiTCN modules, a GRU, a fully connected layer, and an output layer. The whole prediction process is mainly completed by the following steps.

Step 1: The raw data collected by IoT sensors are sliced and preprocessed.

Step 2: The sequences obtained through a sliding window are used as inputs to the model, where each segment is processed by BiTCN’s bi-directional convolution. The fused features from this process then serve as inputs to the GRU.

Step 3: The data from each slide is fed into the BiTCN-GRU model for training, ultimately predicting the waterlogging depth value.

BiTCN, as the core of the whole model, is inspired by Temporal Convolutional Networks (TCN). It employs bi-directional dilated convolution to expand the sensory field, effectively capturing temporal dependencies without increasing computational complexity. The use of residual connections makes BiTCN more stable during training, and the convolution operations can be processed in parallel to improve the training efficiency. The BiTCN designed in this paper consists of a four-layer structure. The first layer consists of two causal dilation convolutions with a convolution kernel size of 3, an expansion factor of 1, and a number of filters of 32. The expansion factors for the second, third, and fourth layers of the network are 2, 4, and 8, respectively, with the rest of the parameters the same as those for the first network layer. The outputs of the BiTCN layer are then reshaped and transferred to the GRU layer to sequentially extract the long-term dependencies. The GRU, due to its unique structure, greatly improves the training efficiency, then two fully connected layers are accessed and finally the output layer outputs the predicted waterlogging depth values.

### 3.2 BiTCN model

**Fig 2:**
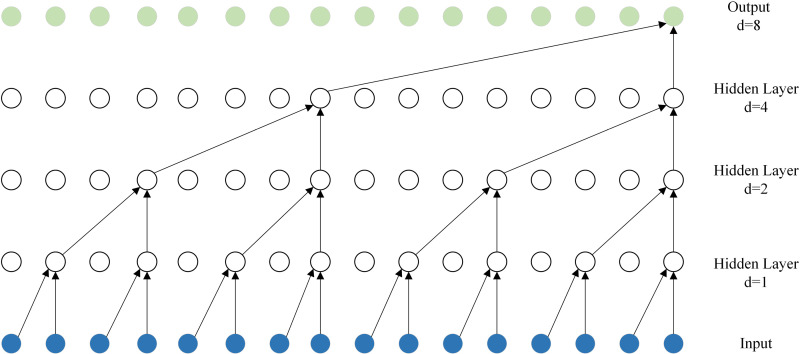
Model structure of dilated convolution.

Proposed by Olivier et al [25], BiTCN uses two bi-directional expansive convolutional networks: one TCN is responsible for encoding the future covariates, while the other is responsible for encoding the past covariates and historical values of the sequence. Thus, the model can learn temporal information from the data and the use of convolution maintains computational efficiency. Bidirectional dilatational convolution, on the other hand, uses the same number of future samples as previous samples for prediction as dilatational convolution. (e.g., **Fig 2**)

**Fig 3:**
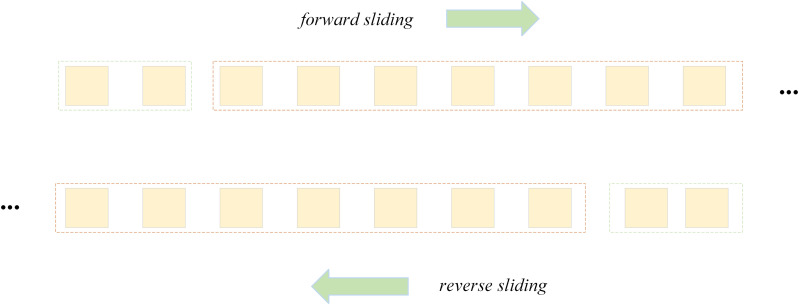
Forward and backward convolution.

What we would like to further illustrate here is that the model contains two independent convolution paths: one deals with the forward information of the time series, i.e., the time-step data from the past to the current; the other deals with the backward information, i.e., the time-step data from the past to the current. The results of these two paths are usually fused at the end and used to predict the output of the current time step. That is, the model can perform forward and backward convolution on the input data. As shown in **Fig 3**.

**Fig 4:**
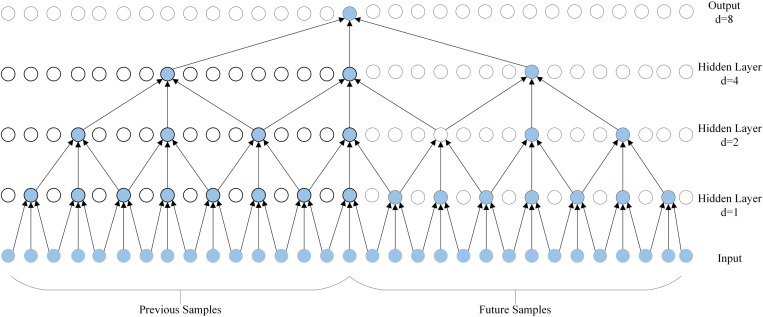
Model structure of bi-directional dilation convolution.

However, bidirectional null convolution can be effective in extracting feature information at the cost of latency because it uses backward convolution, which is what we call data from future moments. The structure of the model when the convolution size is 3 and the expansion factor is [1,2,4,8] is shown in **Fig 4**.

The residual network structure proposed by He et al [30] is a good solution to the problem of gradient vanishing and explosion. Residual connection is to add the features extracted from the model with the original features and short-circuit connection to avoid the problem of losing important original features in the process of information extraction, which is conducive to improving the stability of the model and increasing the depth of the model. The residual connection can be shown in equation (1).


o=Activation(F(x)+x) (1)

where *F(x)* is the residual network and *x* is the input. Through the residual connection, it can effectively prevent the gradient from disappearing and make the neural network more stable. The residual connection is to take the input *x* value and convert the output *F(x)* through a series of modules, and the calculation method of residual connection is shown in Equation (2).


F(x)=h(x)−x(1) (2)

It is assumed that BiTCN consists of a stack of *k* residual blocks, so the output after residual blocks is shown in Equation (3–4):


$$y^{\left(j,k\right)}=[y_{0}^{\left(j,k\right)},...,y_{T}^{\left(j,k\right)}]$$ (3)

$$y_{t}^{\left(j,k\right)}=\sum_{i=0}^{\ker nel-1}(f(i)\cdot y_{t-m\cdot i}^{\left(j-1,k\right)})+y_{t}^{\left(1,k\right)}$$ (4)

*y*^(*j*,*k*)^ is the output of the *j*th layer in the *k*th residual block, *k* is the serial number of the residual block, and *T* is the sequence length.

**Fig 5:**
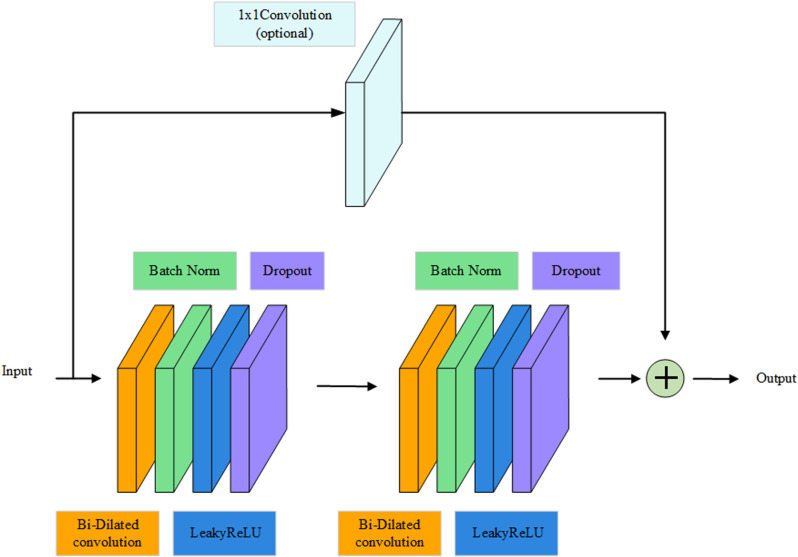
BiTCN residual block.

The BiTCN residual block consists of 2 residual units, and each residual unit consists of Bi-Dilation Convolution, Batch Normalization, Activation Function, Dropout Layer and Residual Connection. Bidirectional dilatation convolution can be used to extract features from both the forward and backward direction of the input data, which enables better extraction of features and better performance of the data between rainfall and waterlogging. Weight normalization through Batch Normalization, normalize the input of the hidden layer to avoid the problem of gradient disappearance, activation function can also help to alleviate the problem of gradient disappearance, the inclusion of Dropout can effectively solve the model overfitting problem, residual connectivity so that deeper layers of the network model performance does not deteriorate. The BiTCN residual block is shown in **Fig 5**.

### 3.3 Gated recurrent unit

**Fig 6:**
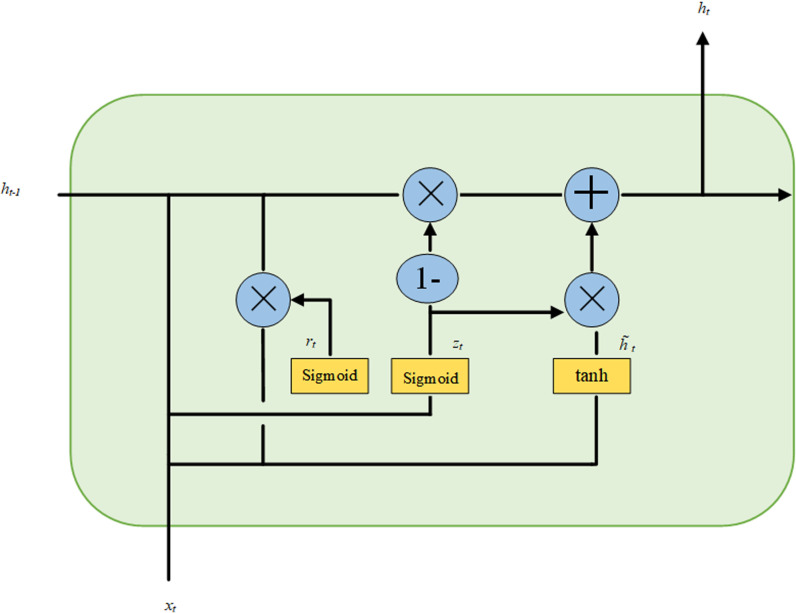
Structure of GRU unit.

GRU is a variant of LSTM. GRU improves the traditional recurrent neural network by solving the problems of gradient vanishing and gradient explosion. Compared with LSTM, the GRU model has a simpler structure and has a better ability than LSTM in network model convergence and parameter update, which leads to a higher computational efficiency of GRU. GRU mainly consists of the following key parts: update gate, reset gate, candidate hidden state at the current moment, and hidden state at the current moment, and the specific structure is shown in **Fig 6**.

### 3.4 Sliding window

The prediction of the depth of waterlogging in urban areas is in fact essentially a time series prediction. Usually, the time series forecasting problem is shown by the following equation (5).


$$Y_{t}=f(Y_{t-1},Y_{t-2},...,Y_{t-n})$$ (5)

*y*_*t*_ is the observation at time *t*, *Y*_*t-1*_*,Y*_*t-2*_*,…,Y*_*t-n*_ are the observations at the previous *n* time points, and ƒ is some function that describes how past observations affect current observations. *Y* is sampled to obtain a series of discrete data points that are distributed equidistant from each other on a timestamp. In this study, the sampling time is set to 5 min. *Y* is a value that varies continuously with t and represents the predicted depth of standing water at future moments. Therefore, the goal of the work in this study is to find a function ƒ from *Y*_*t-1*_*,Y*_*t-2*_*,…,Y*_*t-n*_ to obtain an estimate of the value at the moment.

**Fig 7:**
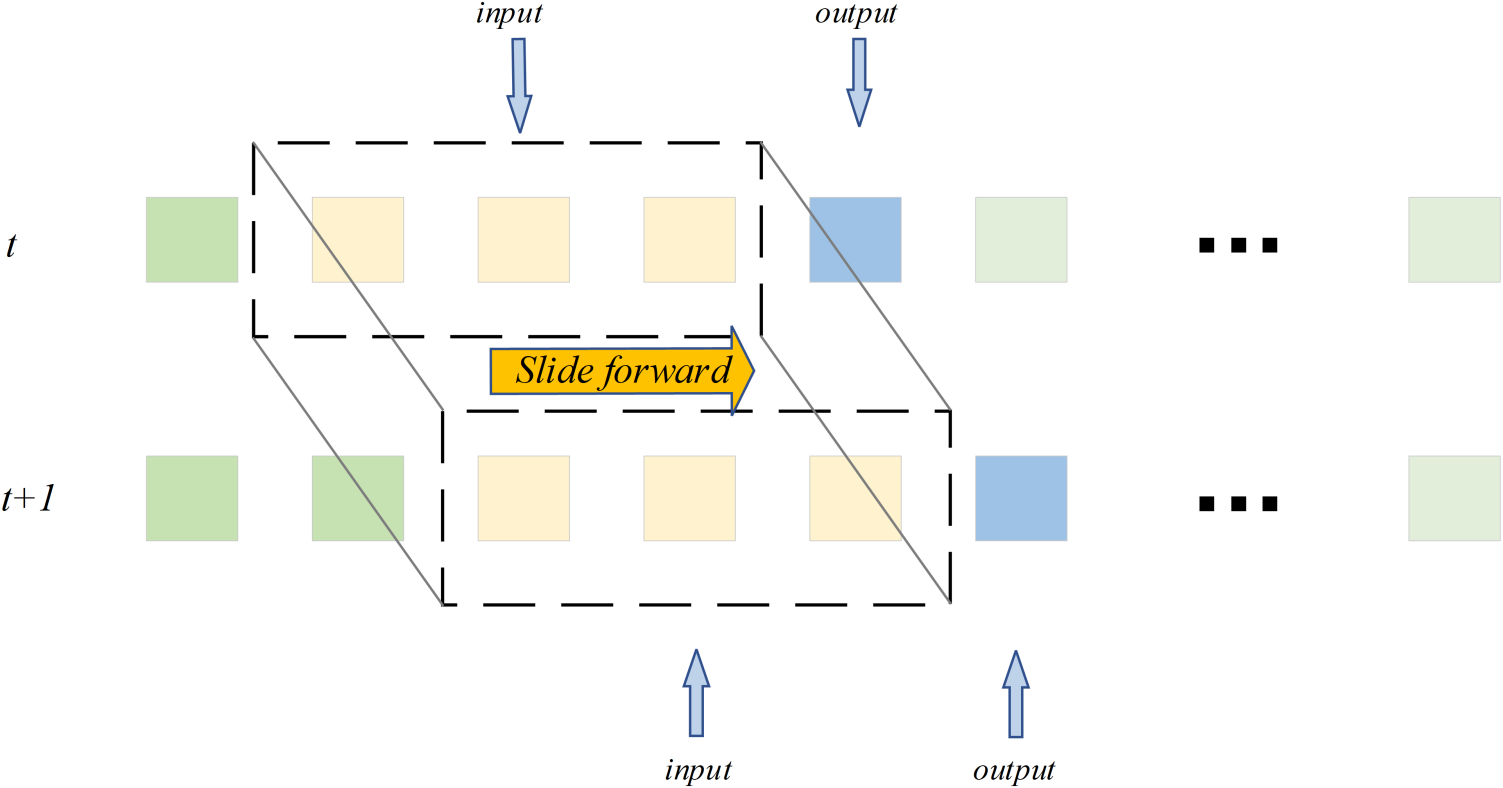
Schematic diagram of sliding process of sliding window.

In this paper, a BiTCN-GRU model fitting function ƒ is used to predict the depth of waterlogging at future moments. Input of model data is achieved by using a sliding window. The schematic diagram is shown in **Fig 7**.

## 4 Experimental results and discussion

### 4.1 Dataset

We use two real datasets to evaluate the accuracy of the BiTCN-GRU model proposed in this paper. The two datasets are sourced from Minshan Road and Huaihe Road, with detection metrics comprising rainfall and water depth, spanning from August 20, 2019 to October 18, 2020, respectively. The original dataset contains data that record a large amount of non-rainfall. The details of the two datasets are presented in **Table 2**.

**Table 2:** Detailed information of the two datasets (Time Interval: 5 minutes).

Dataset	MinshanRoad Dataset	HuaiheRoad Dataset
Total data	26457	17233
Numerical range	0-150mm	0-310mm
Time	2019.8.20-2020.10.18	2019.8.20-2020.10.18

### 4.2 Data preprocessing

To enhance prediction accuracy and real-time performance, we extracted the rainfall and waterlogging data from the raw datasets, employing a sampling period of 5 minutes. Subsequently, we partitioned the preprocessed data in an 80:20 ratio, where 80% of the data was allocated for BiTCN-GRU model training and 20% for BiTCN-GRU model testing.

In addition, data normalization is quite important for rainfall and waterlogging depth. In order to avoid affecting the forecasting results of the BiTCN-GRU model due to the difference in data magnitude values, data normalization operation is required. The formula for data normalization is shown in (6):


$$x^{*}=\frac{x-x_{\min}}{x_{\max}-x_{\min}}$$ (6)

where *x*^ *^ is the normalized value, *x* is the original value of the dataset, and *x*_*max*_ and *x*_*min*_ denote the maximum and minimum values of the data, respectively.

### 4.3 Performance criteria

In this paper, we utilize the root mean square error (RMSE) [31], the mean absolute error (MAE) [32], and the absolute coefficient (*R*^*2*^) [33] to evaluate the performance of waterlogging depth prediction. RMSE is very sensitive to errors in the size of the predicted outcomes and therefore gives a good indication of prediction accuracy. The smaller the value, the better. MAE is used to describe the difference between the predicted value and the true value, again the smaller the value, the better. *R*^*2*^ is a metric used to assess the goodness of fit of a regression model, which indicates the proportion of the data variance that the model is able to explain, and is usually used to compare the performance of different models. Its value ranges from 0 to 1. The formulas are shown as(7–9):


$$RMSE=\sqrt{\frac{1}{n}\sum_{i=1}^{n}{\left(y_{i}-{\tilde{y}}_{i}\right)}^{2}}$$ (7)

$$MAE=\frac{1}{n}\sum_{i=1}^{n}\left|y_{i}-{\tilde{y}}_{i}\right|$$ (8)

$$R^{2}=1-\frac{\sum_{i=1}^{n}{\left(y_{i}-{\tilde{y}}_{i}\right)}^{2}}{\sum_{i=1}^{n}{\left(y_{i}-\bar{y}\right)}^{2}}$$ (9)

where *y*_*i*_ is the true value, ${\tilde{y}}_{i}$ is the predicted value and *n* is the number of predicted values.

The loss function is used to calculate the difference between the predicted value and the true value. In this experiment, in order to improve the prediction accuracy, all the mean square error [34] (MSE) is used as the loss function as shown in Equation (10) to reduce the difference between the true value and the predicted value. The smaller the MSE, the higher the prediction accuracy.


$$MSE=\frac{1}{n}\sum_{i=1}^{n}{\left(y_{i}-{\tilde{y}}_{i}\right)}^{2}$$ (10)

where *n* is the total number of samples, *y*_*i*_ is the true value of the *i*th sample in the data set, ${\tilde{y}}_{i}$ is the predicted value of this sample.

### 4.4 Simulation results and analysis

#### 4.4.1 parametric training.

In this section we present the experimental results and discuss them. The BiTCN-GRU was implemented on a workstation with an Intel Core i5-12490F 3GHz CPU, 16GB RAM and an NVIDIA GeForce RTX 3060 Ti. The experimental environment was mainly based on Python 3.9 under Pycharm version 2021.3 and Tensorflow 2.15 for the implementation. To ensure the accuracy of the experimental results, we maintained consistent model parameters across the same dataset, as variations in model parameter settings can significantly impact overall predictions. This experiment mainly includes short-term prediction of waterlogging depth as well as multi-step prediction in the long-term case.

**Fig 8:**
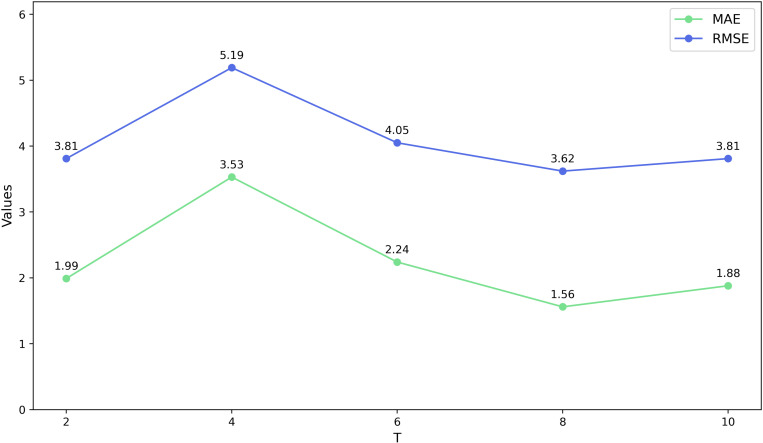
Different time steps in the sliding window on the Minshan Road dataset.

**Fig 9:**
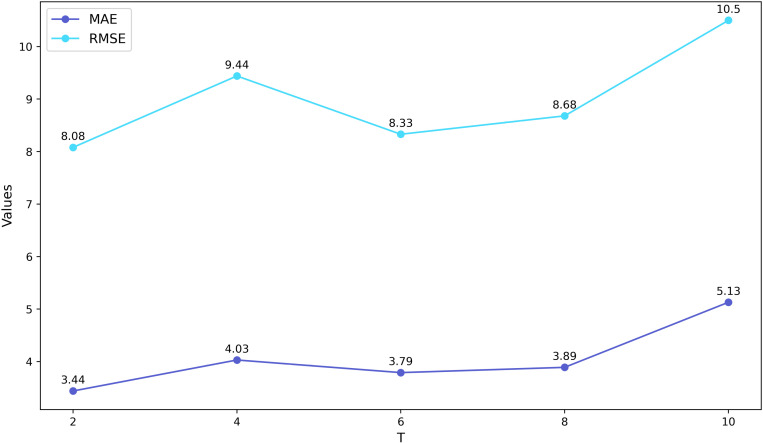
Different time steps in the sliding window on the Huaihe Road dataset.

To investigate the impact of varying time steps in the sliding window on the waterlogging depth prediction model and to determine the optimal time step, we conducted tests on the Minshan Road and Huaihe Road datasets separately. **Figs 8** and **9** illustrate the MAE and RMSE results of the prediction models under different time steps on the two datasets, respectively.

The results indicate that the RMSE of the BiTCN-GRU prediction model reaches the lowest value when *T* = 8 on the dataset of Minshan Road, while the RMSE is minimized at *T* = 2 for the Huaihe Road dataset. Additionally, the other two performance indicators also reach their optimal values under these conditions. Therefore, we select *T* = 8 for the Minshan Road dataset and *T* = 2 for the Huaihe Road dataset for the subsequent experiments.

For optimizer selection, we compare four candidate optimizers, Stochastic Gradient Descent (SGD), Adaptive Incremental (Adadelta), Adaptive Gradient Algorithm (Adagrad) and Adaptive Moment Estimation (Adam). **Table 3** shows the effect of the four different optimizers on the two datasets.

**Table 3:** Comparison of different optimizers on two datasets.

Model	optimizer	Dataset					
		Minshan Road			Huaihe Road		
		MAE	RMSE	*R* ^*2*^	MAE	RMSE	*R* ^*2*^
BiTCN-GRU	SGD	1.86	3.92	86.33%	14.78	16.72	68.50%
	Adadelta	2.69	4.28	83.68%	17.33	25.63	25.97%
	Adagrad	1.69	3.74	87.52%	8.23	11.86	84.13%
	Adam	**1.56**	**3.62**	**88.31%**	**3.44**	**8.08**	**92.64%**

Comparison is made by experimental observation on both datasets. Adam achieves the fastest convergence speed and the lowest loss. Therefore, we choose Adam as the optimizer for both datasets. In addition the number of neurons (*n*) in the appropriate hidden layer has a significant effect on the model. We set a set of values to take different values in [16,32,64,128,256,512], and then select the best hidden layer neuron value n by comparing the prediction results. experimentally, we found that the three metrics reach the best when *n* = 256 on the Minshan Road dataset, and the three metrics reach the best when *n* = 64 on the Huaihe Road dataset.

Through the series of experiments described above, we set the same model parameters on the same dataset, as detailed in **Table 4** below. In addition, for a fair comparison with the baseline model, the same model parameters were used for all baseline models.

**Table 4:** Parameter settings on BiTCN-GRU for Minshan Road and Huaihe River Road.

Parameter	Minshan Road Value	Huaihe Road Value	Description
*T*	8	2	Previous time steps
Optimizer	Adam	Adam	Optimizer
*n*	256	64	Number of hidden layer neurons
Batch size	32	32	Batch size
Learning rate	0.001	0.0001	Learning rate

#### 4.4.2 Ablation study.

In order to validate the effectiveness of the BiTCN-GRU model proposed in this paper, we first compare the BiTCN-GRU model with other widely used baseline models on both datasets. Also, in order to verify the effectiveness of different optimization operations we deeply use visualization operations to show the details of the prediction errors between different models. **Table 5** lists the prediction results of different models.

**Table 5:** Comparison of MAE, RMSE, and *R*^*2*^ for different models.

Model	Dataset					
	Minshan Road			Huaihe Road		
	MAE	RMSE	*R* ^2^	MAE	RMSE	*R* ^2^
BP	1.52	4.05	85.41%	6.58	11.05	86.25%
SVR	3.47	4.52	81.79%	6.93	9.62	89.58%
GRU	2.22	3.71	87.73%	4.92	9.35	90.16%
TCN	1.74	3.86	86.75%	5.43	9.99	88.73%
TCN-GRU	1.53	3.76	87.34%	3.97	8.61	91.64%
BiTCN-LSTM	2.23	4.30	83.53%	3.83	8.51	91.83%
BiTCN-GRU	**1.56**	**3.62**	**88.31%**	**3.44**	**8.08**	**92.64%**

From **Table 5**, it can be seen that the BiTCN-GRU model proposed in this paper achieves better prediction results on both datasets. It proves that the model specifically has a certain degree of generalization. The MAE, RMSE, and *R*^*2*^ of BiTCN-GRU model on the Minshan Road dataset achieve 1.56, 3.62, and 88.31% prediction results, respectively. Compared to BP, SVR, GRU, TCN, TCN-GRU, and BiTCN-LSTM the RMSE improved by 11.8%, 24.9%, 2.5%, 6.6%, 3.9%, and 25.7%, respectively. The *R*^*2*^ improved by 2.9%, 6.52%, 0.58%, 1.56%, 0.97%, and 4.78%. The MAE, RMSE, and *R*^*2*^ of BiTCN-GRU model on Huaihe Road dataset reached the optimal values of 3.44, 8.08, and 92.64%, respectively. Compared to BP, SVR, GRU, TCN, TCN-GRU, BiTCN-LSTM the RMSE is improved by 36.8%, 19.1%, 15.7%, 23.6%, 6.6%, 48.3%, and the *R*^*2*^ is improved by 6.39%, 3.06%, 2.48%, 3.91%, 1%, and 0.81%, respectively. The above data well illustrate the superiority of the BiTCN-GRU model.

**Fig 10:**
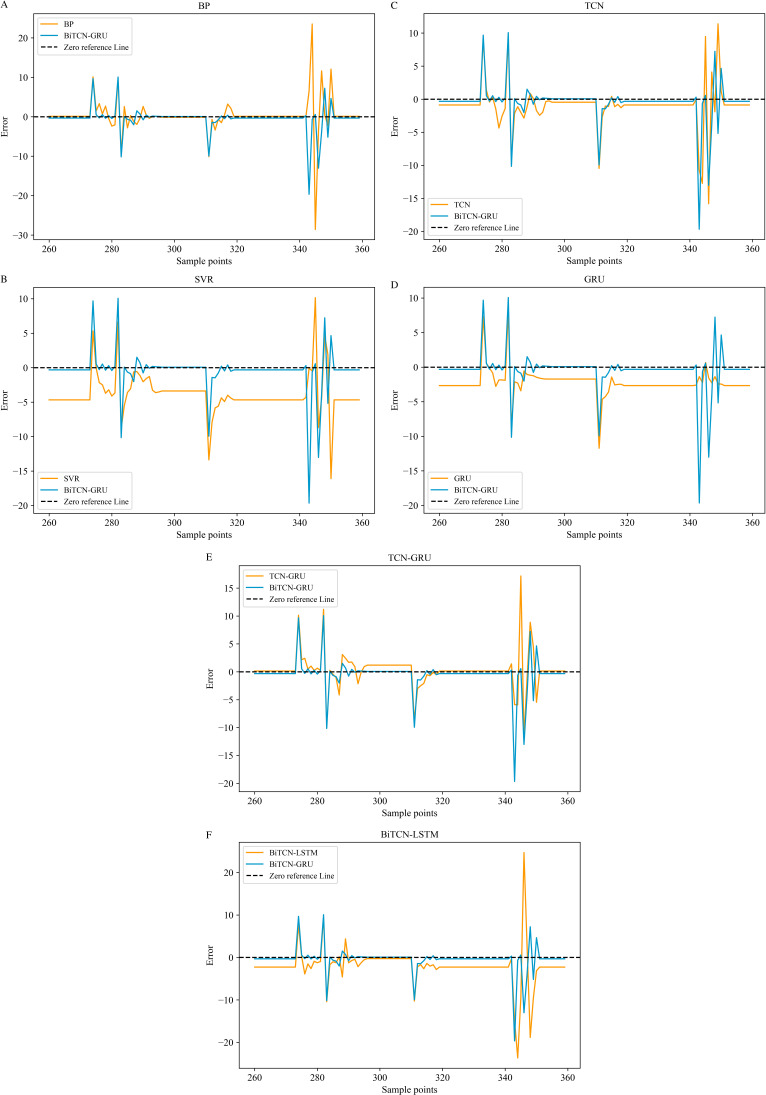
Prediction error plots of different models on the Minshan Road dataset. (a-f).

**Fig 11:**
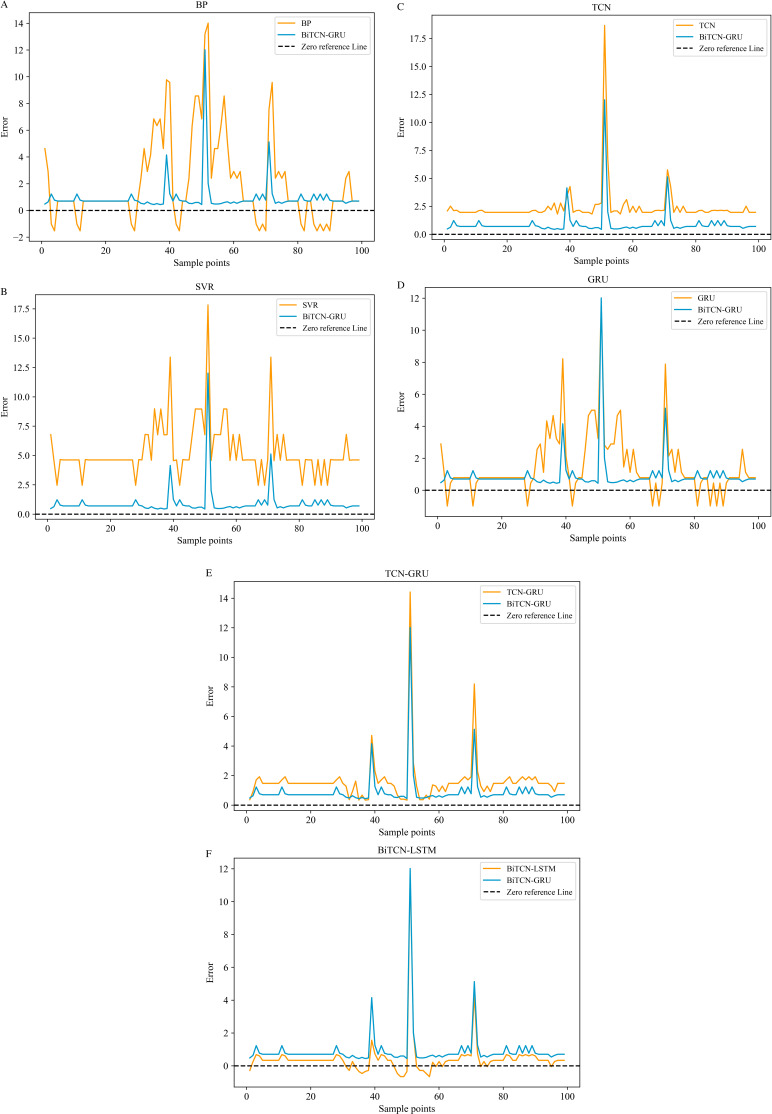
Prediction error plots of different models on Huaihe Road dataset. (a-f).

**Figs 10** and **11** use the visualization of error plots to reflect the prediction performance of each model by selecting the more difficult to predict part of the true value inflection point, which reflects the excellence of the model from different perspectives.

It can be clearly seen from **Figs 10** and **11** that the black dashed line is the zero reference line, and the closer it is to the zero reference line, the better the prediction is. Although all the above seven models can predict the depth of waterlogging, it is not difficult to find that the BiTCN-GRU model proposed in this paper has smaller overall error fluctuations on the two datasets compared to several other modeling algorithms, which are more inclined to the zero reference line. In the process of rainfall and waterlogging, the bidirectional time convolution of BiTCN can effectively extract the features before and after rainfall and waterlogging by forward and backward convolution, which makes its prediction results more accurate compared with other models. However, on the Huaihe Road dataset, as shown in (f) of **Fig 11**, the prediction error is not as good as BiTCN-LSTM, which is mainly because in the overall prediction, there is a phenomenon that the fitting effect is not as good as that of the other models at a certain time node.

#### 4.4.3 Comparative experiments.

To verify the superiority and generalization of the model proposed in this paper, we compare the actual prediction results and various performance metrics of mainstream deep learning algorithms in the field of waterlogging depth prediction, such as GBDT, LSTM, and TCN-LSTM, with those of BiTCN-GRU model, and validate them on the Minshan Road and Huaihe River Road datasets, respectively, and the comparison results are shown in **Table 6**.

**Table 6:** Comparison of BiTCN-GRU with other deep learning models.

Model	Dataset					
	Minshan Road			Huaihe Road		
	MAE	RMSE	*R* ^2^	MAE	RMSE	*R* ^2^
GBDT [25]	4.72	7.15	54.39%	6.85	9.67	89.47%
LSTM [27]	2.25	3.73	87.60%	5.07	9.79	89.19%
TCN-LSTM [28]	1.93	4.15	84.65%	3.61	8.47	91.92%
BiTCN-GRU	**1.56**	**3.62**	**88.31%**	**3.44**	**8.08**	**92.64%**

As can be seen from **Table 6**, all of the above existing methods can achieve the prediction of the depth of waterlogging, but with varying results. The BiTCN-GRU model proposed in this paper achieves better results on both datasets. Compared with the GBDT model, the BiTCN-GRU model proposed in this paper reduces the MAE values by 202.6% and 99.1%, the RMSE values by 97.5% and 19.6%, and the R^2^ directly improves by 33.82% and 3.13% on the Minshan Road and Huaihe Road datasets, respectively. The reason may be that the GBDT model is essentially a static model, which does not directly consider the relationship between data and time series and is not good at capturing the time dependence and long-term trend in time series data, while its handling of lagged data is also deficient, which leads to its ineffectiveness in forecasting. Meanwhile, it is not difficult to observe that the prediction performance of GBDT on Huaihe Road is much higher than that on Minshan Road, and it can be judged that the generalization of this kind of model is not high. The model proposed by Liu and Yao et al. has a great improvement in prediction effect compared to GBDT, which is attributed to the substantial enhancement of the LSTM neural network model’s ability of long-term dependency processing. However, comparing with the BiTCN-GRU model proposed in this paper, there still exists a certain gap, the MAE value of the BiTCN-GRU model is reduced by 44.2% and 47.4% on Minshan Road and Huaihe Road datasets, respectively, the RMSE value is reduced by 3.03% and 2.1%, and the *R*^*2*^ is directly improved by 0.71% and 3.45%, and the main reason for this is that the bidirectional time dilation convolution in BiTCN can effectively capture the sequence features before and after the rainfall and waterlogging process, and secondly, the structure of GRU is simpler and more computationally efficient, thus obtaining higher model accuracy.

**Fig 12:**
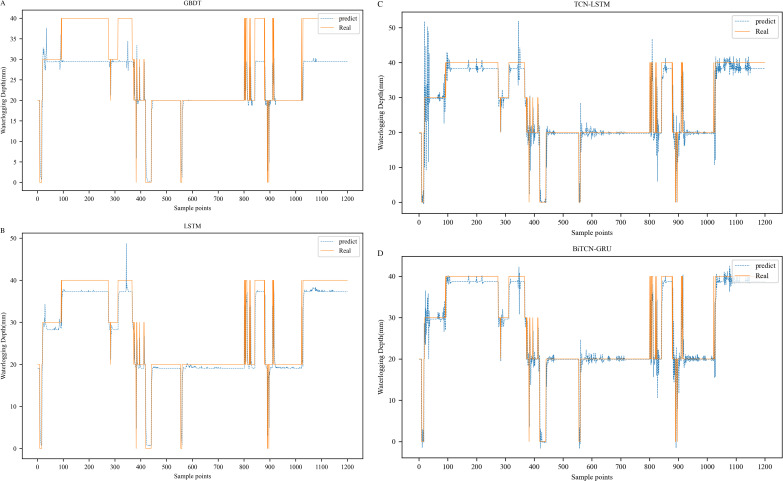
Comparison of prediction results of different models on Minshan Road dataset.

**Fig 13:**
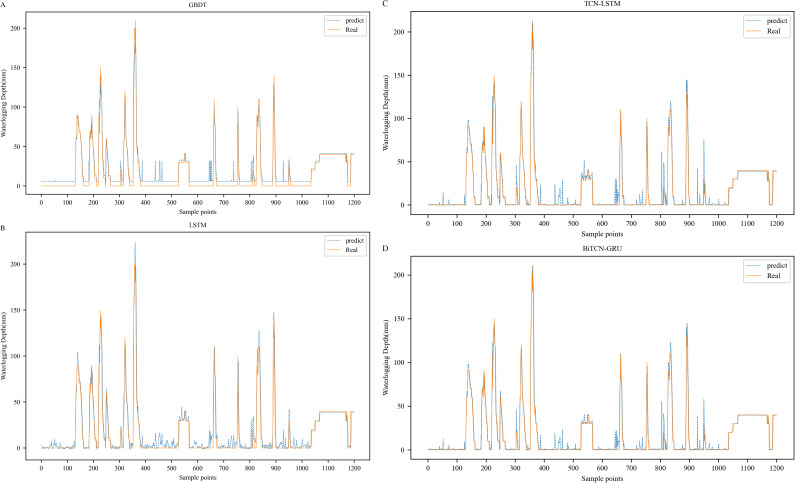
Comparison of prediction results of different models on Huaihe Road dataset.

In addition we visualize the results predicted by GBDT, LSTM, and TCN-LSTM on the Minshan Road and Huaihe Road datasets, respectively. **Figs 12** and **13** show the fitting plots of each model on the Minshan Road and Huaihe Road datasets, respectively.

It can be observed from Figs 12 and 13 that all four models fit the true value curve direction better. However, it is not difficult to find that although all four models can predict the depth of waterlogging values, there are some deviations, and compared with a single model, the fitting effect of the hybrid model is better than that of a single model, which proves that the generalization of the model is stronger. In most of the time periods, the predicted values of the BiTCN-GRU model are closer to the actual values than those of other methods in terms of trend fitting and peak fitting, which proves the superiority of its prediction results. However, in a few inflection points, there are also problems with poorer prediction curves and larger deviations. The BiTCN-GRU model may not perform well in some cases, which may be due to interference from noise in the data or features in the input data that have not been selected or transformed properly, which may result in the model not being able to extract useful information from the data. In summary, our proposed BiTCN-GRU model still achieves the highest prediction accuracy among these comparison models.

#### 4.4.4 Long-term forecasts.

In order to verify the validity of the model in the long-term prediction case, we also performed a multi-step prediction by predicting the long-term prediction for the future for the 2-step (i.e., 10-minute) and 4-step (i.e., 20-minute) scenarios on the two datasets, respectively. The long-term predictions are shown in **Table 7** below.

**Table 7:** Multi-step prediction of BiTCN-GRU on Minshan Road and Huaihe Road datasets.

Model	Prediction Multiple step	Dataset					
		Minshan Road			Huaihe Road		
		MAE	RMSE	*R* ^2^	MAE	RMSE	*R* ^2^
BiTCN-GRU	1	1.25	3.62	88.31%	3.44	8.08	92.64%
	2	1.65	4.18	84.34%	5.12	10.93	85.90%
	4	2.34	4.92	78.08%	7.56	15.41	69.90%

As can be seen in **Table 7**, in the case of a multi-step prediction of 2 steps, the value of the BiTCN-GRU model’s coefficient of determination remains above 80%, indicating that its prediction is quite good. However, for the future prediction of 4 steps, i.e., after 20 minutes, the value predicted by the BiTCN-GRU model obviously begins to deteriorate, and the decision coefficients are directly below 80%. From this, it can be concluded that the waterlogging depth prediction model proposed in this paper has good results for predictions within 10 minutes.

In summary, the BiTCN-GRU hybrid model proposed in this paper is able to achieve prediction in the short and long term and achieves good prediction results on both datasets. The accuracy is good under short-term prediction and the long-term prediction can be effective within 10 minutes with good generalization. This is mainly due to the fact that the BiTCN module effectively extracts the features of rainfall and waterlogging processes, while the GRU is better at handling long-term dependencies. Compared to other models, the BiTCN-GRU model has high accuracy under short-term prediction as well as good real-time performance, which is effective for deep prediction of urban waterlogging.

## 5 Conclusion

Addressing the increasingly severe issue of urban waterlogging caused by the frequent occurrence of extreme weather in recent years, this paper proposes a prediction method for urban waterlogging based on the BiTCN-GRU model. Firstly, the time series data on rainfall and waterlogging depth are collected using IOT sensing devices. To address the low prediction accuracy of the GRU single model, we use BiTCN to extract features from the input data in both forward and backward directions and input the fused extracted features into the GRU. This can fully capture the correlation characteristics of the data before and after the rainfall and waterlogging process. Therefore, based the BiTCN-GRU model proposed in this paper, it is both practical and meaningful for urban waterlogging prediction and early warning systems. It facilitates timely access to information regarding urban waterlogging, thereby reducing the likelihood of disasters and enhancing the safety of citizens.

The dataset we used in this paper has a low feature dimension, and in the future we will enrich the dataset by collecting more features through IoT devices. Therefore, our research will also incorporate feature construction on the dataset and deeper mining of features between the data to improve the prediction accuracy of the model. In addition, we will incorporate weather forecasting to improve the real-time prediction of urban waterlogging.
